# Biomechanical factors in the open gingival embrasure region during the intrusion of mandibular incisors: A new model through finite element analysis

**DOI:** 10.3389/fbioe.2023.1149472

**Published:** 2023-03-29

**Authors:** Yubohan Zhang, Jie Gao, Xu Wang, Jihong Wang, Xu Zhang, Shishu Fang, Wei Wang, Yanning Ma, Zuolin Jin

**Affiliations:** ^1^ State Key Laboratory of Military Stomatology and National Clinical Research Center for Oral Diseases and Shaanxi Clinical Research Center for Oral Diseases, Department of Orthodontics, School of Stomatology, Air Force Medical University, Xi’an, China; ^2^ Xijing Hospital of Digestive Diseases, Air Force Medical University, Xi’an, China; ^3^ The First People’s Hospital of Xianyang, Xianyang, Shaanxi Province, China; ^4^ General Hospital of Southern Theater Command of the Chinese People’s Liberation Army, Guangzhou, China; ^5^ Urumql DW Innovation InfoTech Co., Ltd., Xinjiang, China; ^6^ Stomatological Hospital, Shanxi Medical University, Taiyuan, Shanxi Province, China

**Keywords:** open gingival embrasure (OGE), clear aligners, intrusion movement, bone thickness, inclination, finite element analysis (FEA)

## Abstract

**Introduction:** Open gingival embrasure (OGE) is a common complication in adults following clear aligner therapy and the influence of gingival or alveolar bone biotype on OGE is of great concern. Unfortunately, due to the limited number of patients with clearaligner therapy and the clinical methods to distinguish the gingival biotype of patients being invasive, it is difficult to carry out clinical studies on the gingival or alveolar bone biotype of the OGE. In the meanwhile, the detailed biomechanics of the occurrence of OGE remains unknown. The goal of this study was to establish a new model to simulate the virtual space region, namely, the OGE region, to investigate the relationship between alveolar bone biotype and the occurrence of OGE, and explore potential biomechanical factors related to OGE.

**Methods:** The OGE region in the interproximal space was established using a filler with a very low modulus of elasticity (1 × 10^−6^ MPa). To illustrate the biomechanics of OGE more exhaustively, a line was created at the top of the alveolar crest along the proximal tooth root. FEA was then used to analyze the biomechanics of the surrounding tissues, the OGE region and the line at the top of the alveolar crest along the proximal tooth root of the central incisor under two different labial bone thicknesses (thick and thin) with an axial inclination of 80°, 90° and 100°.

**Results:** During intrusion of the incisors in clear aligner therapy, as inclination increased or bone tissue became thinner, the stress in the surrounding tissues [tooth root, alveolar crest, and periodontal ligament (PDL)] was greater. In the OGE region and interproximal alveolar crest, the strain increased with increasing inclination and labial bone thinning. The results from the line at the top of the alveolar crest along the proximal tooth root showed more detailed biomechanics: In all groups, stress and strain were focused on the mesial-labial alveolar crest. Interestingly, our results also demonstrated that when OGE occurs, other complications may arise, including root resorption and bone dehiscence.

## Introduction

Open gingival embrasure (OGE) is a common complication in adults who have undergone orthodontic treatment; statistics show that more than one-third of such patients experience OGE after orthodontic treatment (Kurth and Kokich, 2001). In addition to being unesthetic, OGE may cause harm to the periodontium by causing chronic food retention (Kurth and Kokich, 2001). Clear aligners are becoming increasingly popular due to their excellent aesthetic value, although these aligners are also associated with aesthetic problems such as OGE. In our previous study, we found the incidence of OGE in non-extraction patients after clear aligner therapy was relatively high at 25.7% and 40.3%, respectively, between the maxillary and mandibular central incisors (Zhang et al., 2023). The biomechanics of clear aligners differ from those associated with fixed appliances. Fixed appliances exert a continuous light force by arched wires, while clear aligners apply instantaneous and excessive initial stress which may result in pathological reactions (Li et al., 2016; Zheng et al., 2017; Ke et al., 2019). Little is known about the effects of clear aligners; indeed, previous studies were all clinical studies on OGE (Kurth and Kokich, 2001; An et al., 2018), unfortunately, there are no reports in the existing literature that discuss the specific mechanisms underlying the occurrence of OGE.

Intrusion movement is directly or indirectly involved with OGE due to the susceptibility for calculus in the mandibular anterior teeth and the concentration of stress or strain upon the alveolar crest (Melsen, 1986; An et al., 2018; Ma and Li, 2021). In addition, incisor inclination may affect the generation of alveolar defects (Yu et al., 2009). Importantly, it is important to note that post-treatment inclination of the mandibular anterior teeth varies according to the treatment protocol. Compared with patients undergoing extraction treatment plans, the change of anterior tooth inclination in non-extractive patients before and after treatment is limited. Patients with non-extraction treatments might experience a labial tilt of their mandibular teeth. Furthermore, the incidence and severity of OGE in the mandible are higher and more severe than in the maxilla (An et al., 2018). Therefore, when considering OGE, it is vital that we investigate intrusion movement of different axial inclinations in the mandibular anterior teeth.

For many years, scholars have debated whether gingival or periodontal diseases are more likely to occur in patients with a thin gingival biotype (Claffey and Shanley, 1986; Olsson and Lindhe, 1991). However, very little clinical research has addressed the association between the thickness and biotype of the gingiva with OGE. Interestingly, previous work detected positive associations between gingival thickness and labial bone thickness (Fu et al., 2010; Zweers et al., 2014). We hypothesized that bone thickness is not only a biological factor that limits tooth movement by boundaries; in addition, we believe that bone thickness also affects the biomechanics of tooth and bone tissue during intrusion. The association of bone thickness with OGE has yet to be demonstrated in clinical studies due to difficulties associated with case collection; in the present study, we used finite element analysis (FEA) to address this key shortfall in knowledge.

FEA is a valuable tool that can be used to investigate the virtual biomechanics of potential clinical outcomes. The reliability of this method is determined by its design and the specific mechanical properties of tissues and appliances. Some researchers have suggested that predicted displacements and the distribution of the stress field could be affected if non-linear mechanical properties of the PDL were introduced (Hohmann et al., 2011); thus, a more sophisticated representation is warranted (Toms and Eberhardt, 2003). Non-linear mechanical properties of the PDL for orthodontic force have been assigned in few studies.

The relationship between labial alveolar bone thickness and the occurrence of OGE and the underlying mechanical mechanism of OGE remains unknown. Therefore, the aim of this study was to investigate the roles of labial bone thickness (thick alveolar bone and thin alveolar bone) and inclination (incisal mandibular plane angle (IMPA) of 80°, 90° and 100°) in terms of stress and strain distribution around supporting tissues of the mandibular anterior teeth during intrusion movement through FEA. We also aimed to identify and characterize potential biomechanical factors that might affect the occurrence of OGE.

## Materials and methods

### Establishment of the mandible model

Cone beam computed tomography (CBCT) data (HiRes3D-Plus; Largev, Beijing, China) were acquired from a healthy volunteer with well-aligned dentition. Three-dimensional base models of the mandibular dentition were then established using MIMICS version 10.0 software (Materialise, Leuven, Belgium). Optimization and surface model creation were achieved by GEOMAGIC Studio 2014 (Raindrop GEOMAGIC, North Carolina, United States). Then, NX1911 software (Siemens, German) was used to generate a preliminary model for the PDLs by extending the outer surfaces of tooth roots outwards by 0.25 mm. The mandible was moved inwards by 1.3 mm using the offset command and then cortical bone and cancellous bone models were established by Boolean subtraction operation instructions. The study was approved by the Ethical Committee of the Stomatological Hospital of the Air Force Military Medical University.

Researchers previously reported that canine and premolars were subjected to 0.57 N and 0.07 N extrusive forces during the intrusion of incisors, and canine acted as the main form of anchorage (Liu and Hu, 2018). In the present study, we only analyzed the biomechanics of the central incisor. To simplify the model, vertical rectangular attachments (3 mm in height, 2 mm in width, and 1 mm in thickness) were established in the canine as a retaining attachment. The mandibular teeth crown and attachment were then extended outwards by 0.5 mm to simulate the thickness of the appliance, and each tooth was treated as an independent component. All components were imported into ANSYS Workbench 2019 (Ansys, Pennsylvania, United States) to generate a three-dimensional (3D) finite element model for finite element analysis.

The labial bone thickness of the thick-bone biological type was 0.3 mm thicker than that of the thin-bone biological type (Rossell et al., 2015; Shafizadeh et al., 2022). Since the OGE region is composed of the space between two adjacent teeth, it cannot be represented by specific tissue. Consequently, a filler with a very low modulus of elasticity (1 × 10^−6^ MPa) was established in the proximal space of the central incisor; this was used to simulate the OGE region. The strain of the OGE region and the corresponding alveolar crest reflected the degree of alveolar crest absorption and the changes of OGE region and was calculated by FEA. To illustrate the biomechanics of OGE in more detail, a line at the top of the alveolar crest along the proximal central incisor tooth root was established, which could reflect the details of the biomechanical changes from labial to lingual side.

In our study, we considered it important that we assigned the PDL with non-linear material properties in FEA (Table 1; Table 2) (Toms and Eberhardt, 2003). Other structures were assumed to be linear elastic isotropic and homogeneous materials. The mechanical properties were determined by methods described in previous studies, as shown in Table 1 (Ma and Li, 2021).

**TABLE 1:** Properties of the materials considered in this study.

Material	Young’s modulus, MPa	Poisson ratio
Cancellous bone	1.37 × 10^3^	0.3
Cortical bone	1.37 × 10^4^	0.26
Tooth	1.96 × 10^4^	0.3
PDL (linear elastic)	0.143	0.45
PDL (non-linear elastic)	Calculated, see Table 2	0.45
Aligner	528	0.36
Attachment	12.5 × 10^3^	0.36
OGE region	1 × 10^−6^	1 × 10^−3^

PDL: periodontal ligament.

OGE: open gingival embrasure.

**TABLE 2:** Piecewise linear mechanical properties describing the non-linear elastic stress-strain behavior of PDL (values were imported into the FEA model).

Strain, mm/mm	Stress, MPa
0.0000	0.0000
0.1963	0.0019
0.2327	0.0043
0.2704	0.0098
0.2782	0.0115
0.3017	0.0187
0.3342	0.0359
0.3509	0.0497
0.3680	0.0690
0.3908	0.1057

### Configuration settings

**FIGURE 1:**
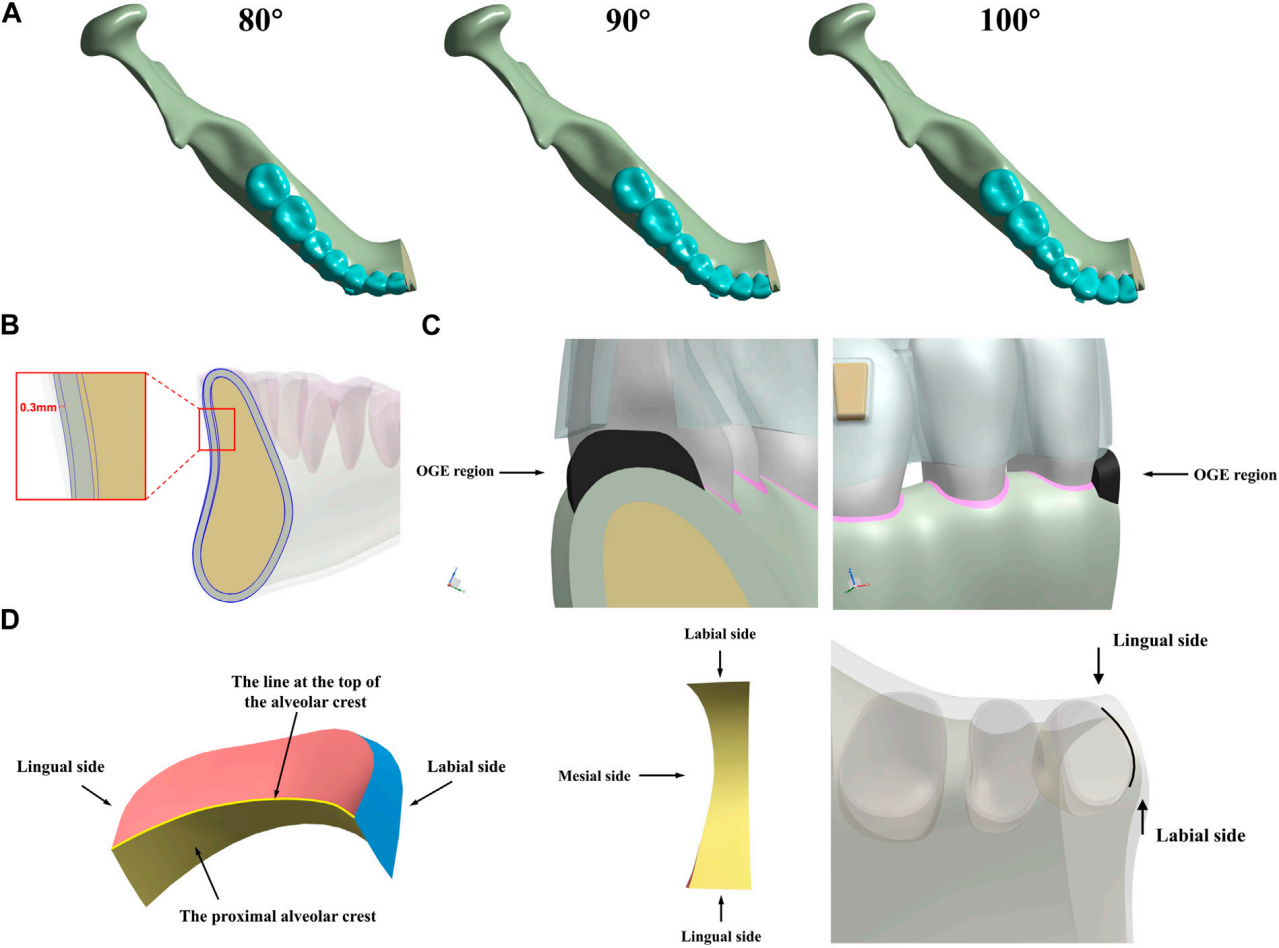
Three-dimensional (3D) mandible models for intrusion movement in incisors. **(A)** different inclinations (80°, 90°, and 100°), **(B)** labial bone thickness biotype (thick, thin) and **(C)** the OGE region, **(D)** different surfaces in OGE region and the line at the top of the interproximal alveolar crest along the root of central incisor.

In Figure 1, two protocols were established according to the labial alveolar bone thickness (configuration 1: thick; configuration 2: thin). Each protocol involved three groups according to different inclinations (IMPA) of the lower incisors (80°, 90°, and 100°).

### Simulation of anterior teeth intrusion

To simulate the intrusion of incisors in mandibular anterior teeth in six models (Thick-80°, Thick-90°, Thick-100°, Thin-80°, Thin-90°, Thin-100°, respectively), the displacement of 0.25 mm was preset (Liu and Hu, 2018) along with the long axis in the apical direction of incisors under three different axial inclinations of anterior teeth (80°, 90°, and 100°) and different alveolar thicknesses. The specific methods are as follows: According to the original dentition model, the incisor teeth were intruded 0.25 mm along the long axis to generate a new dentition model, and the corresponding clear aligners were generated according to the new dentition model. When the clear aligners are placed on the original dentition model, the clear aligners deform and exert intrusion force on the incisor teeth. The lower central incisor was the main observation object in our study.

## Results

**FIGURE 2:**
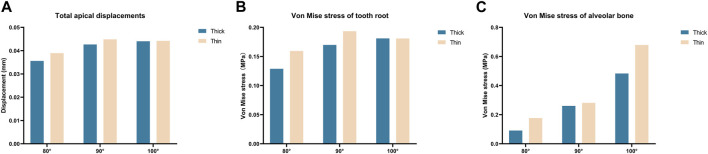
Maximum total tooth displacements, maximum von Mise stress on the tooth root and alveolar bone of the central incisor in various models (Thick-80°; Thick-90°; Thick-100°; Thin-80°; Thin-90°; Thin-100°). **(A)** Maximum total apical displacements, **(B)** maximum von Mise stress of the tooth root, and **(C)** maximum von Mise stress of the alveolar bone.

**FIGURE 3:**
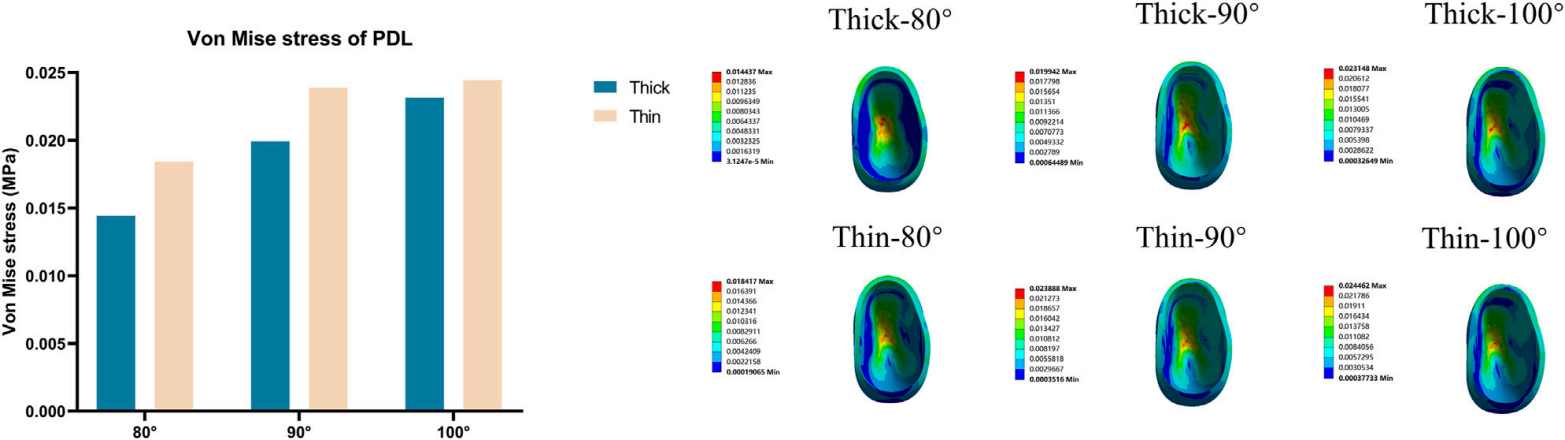
Von Mise stress on the PDL of the central incisor in various models (Thick-80°; Thick-90°; Thick-100°; Thin-80°; Thin-90°; Thin-100°).

As illustrated in Figure 2, we quantified tooth displacement and stress values under two different labial alveolar bone thicknesses (thin; thick) in three different axial inclinations (80°, 90°, and 100°). Following the 0.25 mm intrusion along the long axis of the central incisor, the total displacement of the central incisor in the apical area was shown in Table 3. The von Mises stress value of the thin biotype was stronger than the thick biotype when the anterior teeth were oriented in the same axial direction. Furthermore, as the axial inclination increased, the level of stress also increased (Figures 2, 3). Analysis demonstrated that the stress values of the tooth root, the alveolar bone and the PDL were shown in Table 3.

**TABLE 3:** Tooth displacement, von Mise stress of the tooth root, the alveolar bone and the PDL of the central incisor in various models (Thick-80°; Thick-90°; Thick-100°; Thin-80°; Thin-90°; Thin-100°).

Various models	Tooth displacement, mm	Von mise stress of the tooth root, MPa	Von mise stress of the alveolar bone, MPa	Von mise stress of the PDL, MPa
Thick-80°	0.036	0.129	0.09	0.014
Thick-90°	0.043	0.170	0.261	0.020
Thick-100°	0.044	0.181	0.483	0.023
Thin-80°	0.039	0.160	0.177	0.018
Thin-90°	0.045	0.193	0.282	0.024
Thin-100°	0.044	0.181	0.679	0.024

**FIGURE 4:**
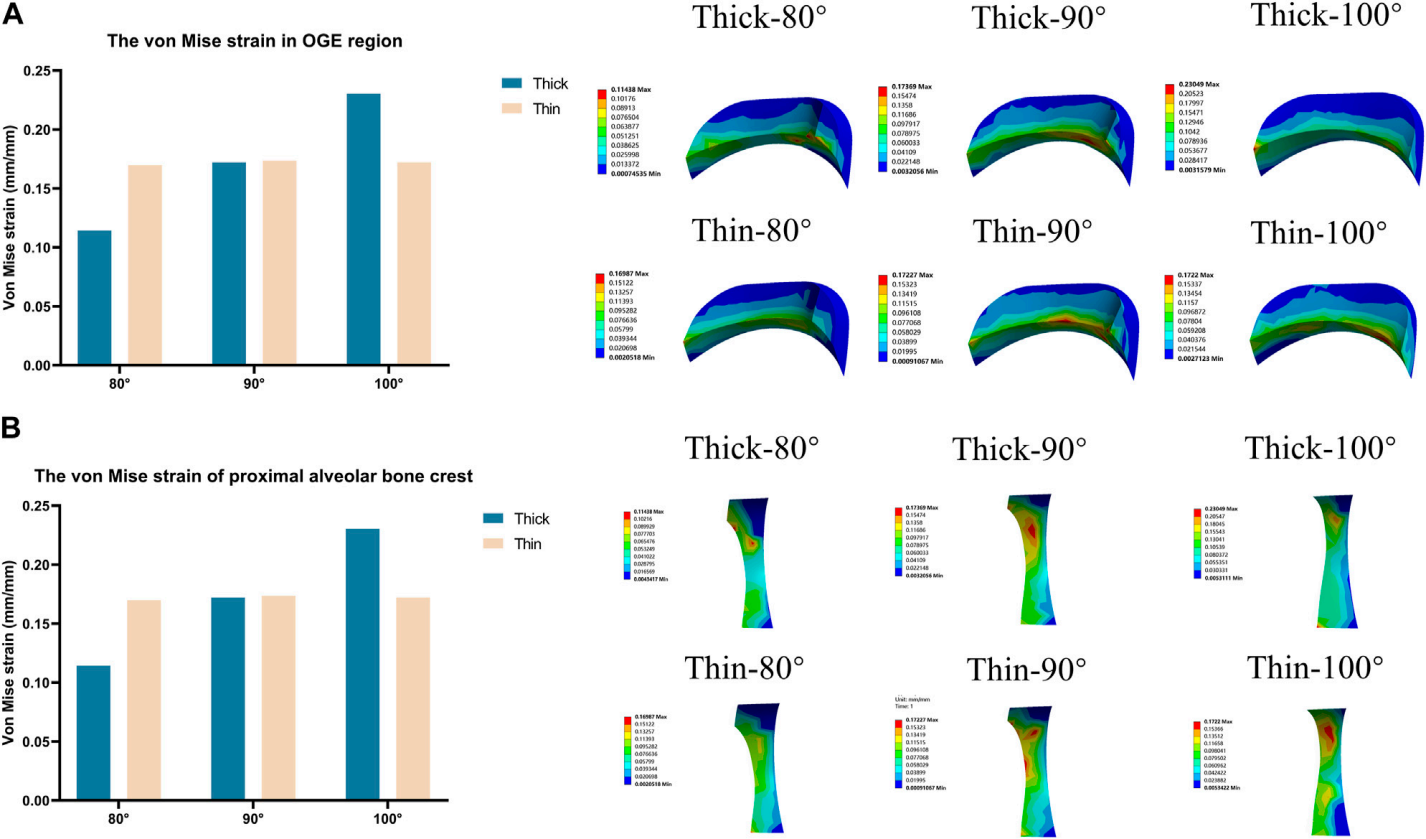
Von Mise strain on the OGE region in various models (Thick-80°; Thick-90°; Thick-100°; Thin-80°; Thin-90°; Thin-100°). **(A)** Maximum von Mise strain in the OGE region, **(B)** maximum von Mise strain on the proximal alveolar bone crest.

The strain values in the OGE region and corresponding alveolar crest with three different axial inclinations in the anterior teeth are shown in Figure 4. Generally, the alveolar ridge crest at the mesial aspect was seriously deformed as the inclination increased or the labial bone thickness became thin. In both thick and thin bone biotypes, the stress was concentrated on the labial side of the alveolar bone crest, which shows that the biomechanics underlying resorption of the interproximal alveolar crest was different on the labial and lingual sides.

**FIGURE 5:**
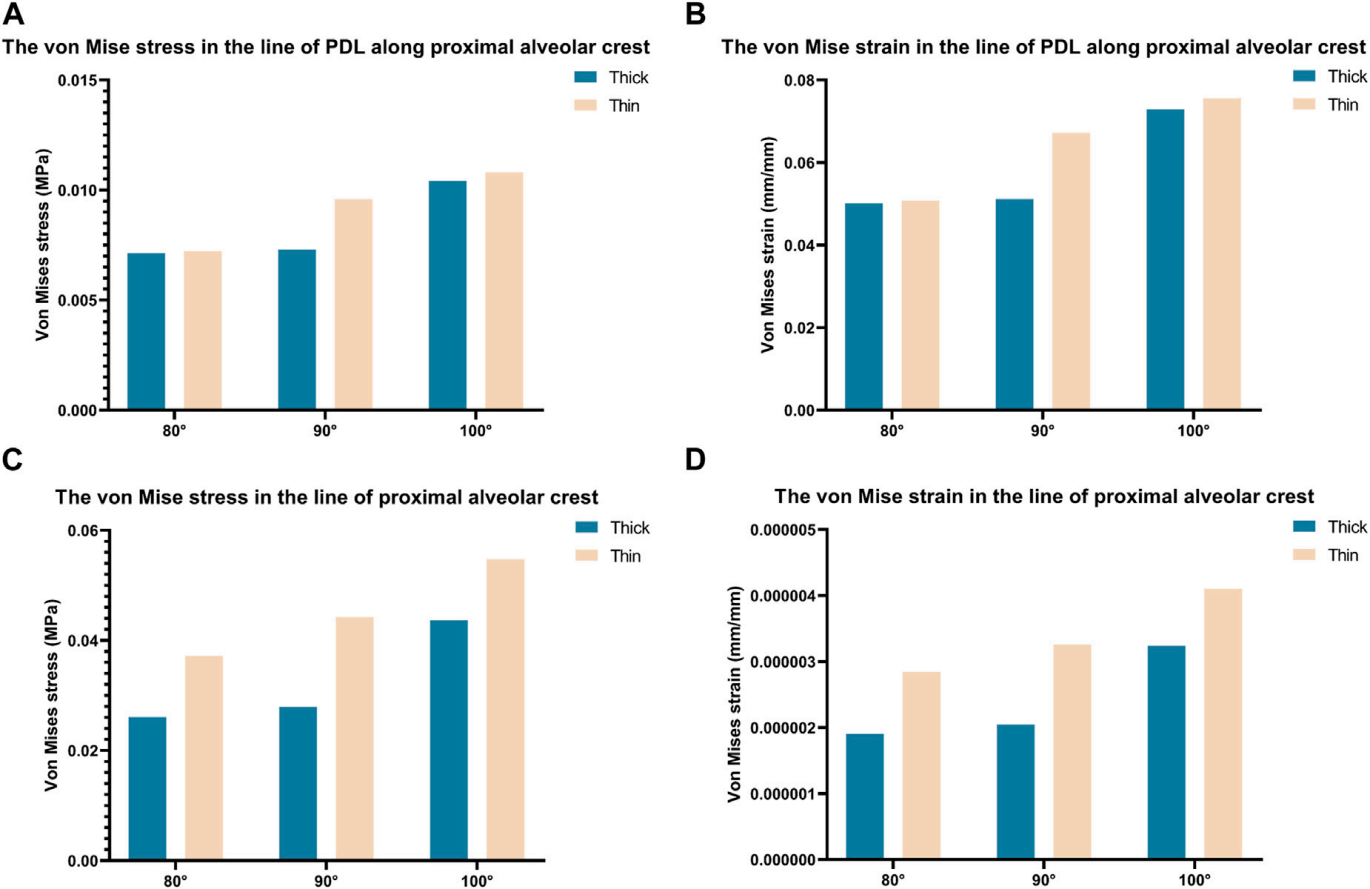
Bar chart of maximum von Mise stress and strain along a line generated along the proximal alveolar crest in various models (Thick-80°; Thick-90°; Thick-100°; Thin-80°; Thin-90°; Thin-100°).

**FIGURE 6:**
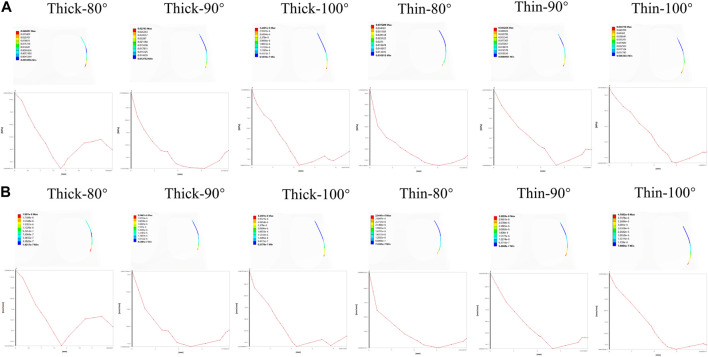
The distribution of von Mises stress and strain on a line generated along the proximal alveolar crest in various models (Thick-80°; Thick-90°; Thick-100°; Thin-80°; Thin-90°; Thin-100°). **(A)** Von Mises stress on the alveolar bone crest, **(B)** von Mises strain on the alveolar bone crest.

To illustrate the biomechanics of OGE in more detail, we created a line at the top of the alveolar crest along the proximal tooth root to see the differences in the biomechanics of the labial and lingual side. The von Mise stress and strain in the PDL and alveolar bone crest along the line in the proximal of lower central teeth were observed in Figure 5. The stress and strain of the alveolar crest in the biological type of thick bone were generally smaller than that of thin bone, as the labial inclination increased, the extent of stress and strain also increased. The distribution of stress and strain of the alveolar crest were shown in Figure 6. The extent of stress and strain in both the thick and thin biotypes were concentrated in the mesial-labial alveolar crest. A line chart was generated and showed the quantification of von Mises stress and strain of the line at the top of the alveolar crest along the proximal tooth root from the labial to the lingual side. The maximum value of stress and strain of the alveolar crest were all focused on the labial side.

## Discussion

The occurrence of OGE is associated with a variety of functional complications, including gingival inflammation, poor gingival health, and the loss of integrity (Burke et al., 1994). In patients with mild-to-moderate malocclusions, clear aligners are an effective alternative to fixed appliances, but not in patients with severe malocclusions (Yassir et al., 2022). As a result, non-extraction patients make up the majority of orthodontic patients undergoing invisible aligner treatment. Based on the application of the finite element model in oral biomechanics and orthodontics, we employed a 3D-finite element model to investigate the biomechanics of OGE. In our study, we induced intrusion on the incisors and found that as the inclination angle increased or the labial bone tissue became thinner, the levels of the stress in the surrounding tissues of the lower central tooth increased. With regards to the OGE region, in the interproximal alveolar bone crest, the strain was mainly concentrated in the mesial-labial region, and the extent of stress and strain was greater on the central incisor with labial inclination and the thin bone biotype.

With low orthodontic forces, the material properties of bone exert only a minor effect on initial tooth mobility; this allows us to focus on the PDL material properties when investigating the intrusion movement in clear therapy (Hohmann et al., 2011). The hydrostatic pressure of the PDL shown in Figure 3 can be used to predict root resorption (Hohmann et al., 2009); in the present study, we observed stress concentration occurred in the root apical region of all groups. Short-term tooth movement is regarded as being primarily governed by PDL deformation (Connizzo et al., 2021). Studies indicated that initial tooth movement is highly dependent on the mechanical properties of the PDL (Middleton et al., 1996; Bourauel et al., 1999; Qian et al., 2008). Based on linear elastic models, tooth displacements have generally been overstated when compared to non-linear elastic models (Shokrani et al., 2020). Non-linear mechanical properties of PDL substantially alters predicted biomechanical outcomes, not only the predicted displacements but also the stress or strain distribution (Hohmann et al., 2011). In order to acquire a more realistic distribution of stress and strain which better can better characterize orthodontic tooth movement, the non-linear material properties of PDL were conducted in our study.

Our analysis showed that stress values were reduced and distributed more symmetrically when teeth were tilted lingually; this finding was also reported in another study (Gerami et al., 2016). Yared found that final inclination (>95°) led to greater and more severe gingival recession on the mandibular central incisors (Yared et al., 2006). In addition, orthodontists should be more cautious about anterior teeth with labial inclination during intrusion in the mandible, especially the incisor (Dorfman, 1978; Artun and Krogstad, 1987; Wehrbein et al., 1996; Evangelista et al., 2010). In the present study, we intruded the incisors along the tooth long axis to mimic a real clinical situation as far as possible, even if the teeth were not within a reasonable window of inclination (80° or 100°); this is crucial because inclination of the mandibular incisors can’t change enough to reach the final ideal position in the non-extraction protocol of treatment. Thus, it is unrealistic to adjust the axial inclination of incisors first and then cause intrusion according to the ideal situation in some cases. Furthermore, the intrusion movement through cumulative reaction of the teeth against vertical forces and tipping movement should be chosen carefully (Melsen, 2001).

For maximum tooth movement efficiency and minimal root and periodontal damage, orthodontic forces should be applied in an appropriate manner (Rossini et al., 2015). Stress (or strain) above a certain threshold could cause pathological bone resorption in bones (Sugiura et al., 2000; Frost, 2003). In a previous study, Lee et al. suggested that the stresses generated were within the optimal range (0.015–0.26 MPa) (Lee, 1965). As shown in Figure 2, the stress values in alveolar bone (at inclination angles of 80° and 90°) were relatively low but then increased notably (Thick-100: 0.483 MPa, Thin-100: 0.679 MPa) with axial inclination of 100° with stress values far beyond the optimal range (Lee, 1965). Researchers found that when the hydrostatic pressure of the PDL reaches 0.005 MPa, bone resorption occurs, thus inducing tooth movement (Hohmann et al., 2009; Chen et al., 2014; Liao et al., 2016). PDL necrosis and dysfunction is considered to occur at a hydrostatic pressure exceeding 0.016 MPa; this is higher than systolic pressure in humans (Liao et al., 2016). As the angle of inclination increased, the stress on the PDL at the root apex increased rapidly (as shown in Figure 3); this increasing trend was more obvious in the thin bone biotype. This gave us a hint that anterior teeth with labial inclination and the thin labial bone biotype are more susceptible to root resorption.

The stress values in tooth roots, the PDL and alveolar bone were higher in the thin bone biotype when compared with the thick bone biotype, this also confirmed that bone thickness not only limited tooth movement as boundaries, but also affected tooth and bone biomechanics. In the OGE region, we found that as the thickness of the labial bone decreased and the angle of inclination increased, the proximal alveolar crest around the tooth endured increasing levels of strain (Figure 4). It is imperative to better understand how stress and strain distribution causes alveolar bone loss in the interproximal area. In the present study, we found that it was possible to acquire further information by generating a line at the top of the alveolar crest along the proximal tooth root, as shown in Figure 6. Interestingly, we observed that stress and strain were concentrated on the mesial-labial side. In other words, in the interproximal region, bone tissue was absorbed from the labial side. Clinically, gingival recession is always accompanied by alveolar bone dehiscence (Dorfman, 1978; Richman, 2011). In a previous study, Steiner et al. showed that when mandibular incisors were moved to the labial side by 3.05 mm, the height of the labial alveolar bone decreased by 5.48 mm (Steiner et al., 1981). Another study also showed that the highest concentration of stress was found in the alveolar bone crest and not in the root apex when intrusive force was applied to a model of healthy alveolar bone (Gupta et al., 2020). When combined with the results shown in Figures 3, 4, 6, these findings also alert clinicians to pay attention to the susceptibility to root resorption and alveolar bone dehiscence when patients are susceptible to OGE during or after orthodontic treatment. These findings are consistent with those of previous studies in that intrusive movement is highly susceptible to apical root resorption, a reduction of alveolar bone height and OGE (Costopoulos and Nanda, 1996; Han et al., 2005; An et al., 2018). Therefore, we postulate that complications associated with orthodontic treatment are interconnected. In clinical practice, when one complication is found, attention should also be paid to the possibility of other complications.

Bone and soft tissue augmentations are mostly performed on the labial or buccal side of the alveolar bone (Jung et al., 2018); these methods could be effective for the treatment of OGE. In addition to the ease and maturity of operation, from another perspective, the effect of OGE and alveolar bone dehiscence on the aesthetic impact was mainly reflected on the labile or buccal side. In our study, the biomechanics were different from labial side to lingual side (Figure 6). Clinically, we can only observe the occurrence of the OGE from the labial side of patients, so it may be ignored that the clinical manifestations of the OGE, bone resorption degree and the morphology (height and thickness) may be different on the labial and lingual side.

Gingival dehiscence may indicate a loss of the underlying vestibular bone (Wehrbein et al., 1994). It is generally believed that bone height is related to the occurrence of OGE. In a previous study, the papilla was detected almost 100% of the time when the distance between the contact point and the crest of the bone was less than 5 mm (Tarnow et al., 1992). The morphology of the alveolar bone constitutes a limiting factor for orthodontic movement, although the pretreatment bone height is not the only factor that should be considered. Lund found that alveolar bone surfaces with the largest cemento-enamel junction (CEJ)-marginal bone crest (MBC) distances at baseline did not show greater changes during orthodontic treatment than surfaces with a smaller CEJ-MBC distance at baseline (Lund et al., 2012). Our findings suggest that labial bone thickness may also be related to the occurrence of OGE in addition to bone height, although this needs to be confirmed by clinical trials.

Understanding the association between gingiva biotype and OGE can facilitate our approach to clinical practice. Because of the difficulties associated with clinical trials, we established finite element models of different alveolar bone thicknesses to simulate different gingival biotypes. It has been documented that labial inclination and thin labial bone thickness can cause gingival recession and that gingival recession could also be associated with OGE and alveolar bone dehiscence (Castro et al., 2016). Labial bone thickness is an important factor that affects resorption which can also cause shrinkage of the soft tissue (Esposito et al., 1993). Biological and biomechanical factors are closely related. The biomechanics of bone thickness has been widely studied in the field of implants but not with regard to clear aligners and the occurrence of OGE. Buser et al. (2004) recommended that the labial bone thickness should be at least 2 mm around the implant; this thickness of alveolar bone on the labial side was recommended to ensure appropriate soft-tissue support and minimize the risk of peri-implant gingival recession (Holmes and Loftus, 1997; Petrie and Williams, 2005). Other researchers proposed that a labial bone thickness of approximately 2 mm could reduce the incidence and extent of vertical bone loss (Qian et al., 2009). However, the optimal threshold for labial bone dimensions could not be determined with regard to achieving the best aesthetic outcome and stress distribution (Teughels et al., 2009). In our study, the thick groups had a bone thickness that was 0.3 mm larger than the thin groups. Interestingly, small increases in labial bone thickness also led to large biomechanical changes. Previous researchers found that cancellous or cortical bones can carry a greater load as their diameter increases (Alikhasi et al., 2014). Similarly, in our study, the strain on the OGE region reduced when the bone thickness increased. These results may also explain the biomechanical changes caused by inclination. Lingual inclination can increase the thickness of the labial bone of the tooth neck; thus, intrusion movement can be more physiological due to the lower and more uniform distribution of stress in the periodontium. In general, our present analysis concurs well with previous studies in that an increase in labial bone thickness was associated with stress reduction (Holmes and Loftus, 1997; Tada et al., 2003).

In a previous study, Yared et al. (2006) found that a final inclination >95° resulted in greater and more severe gingival recession on the mandibular central incisors. Nevertheless, when comparing thickness to the final inclination, it was found that thickness had greater relevance to recession. Notably, the cortical plate was restored virtually to its original thickness after the reverse movement of a root that had already perforated it (Wainwright, 1973; Wehrbein et al., 1994). The results of our current study suggest that patients with mild OGE can be treated, at least to some extent, by lingual inclination within the limits of bone morphology. In addition to adjusting normal axial inclination and stress patterns, the motion of lingual inclination was associated with labial plate remodeling which could increase the thickness of labial bone.

The high prevalence of alveolar bone defects can’t explain the low prevalence of gingival recession before orthodontic treatment (Evangelista et al., 2010). This suggests that soft tissue plays a more important role in the pathogenesis of OGE than bone tissue. Moreover, the thicker the periodontal tissue, the less likely there are to be clinical changes in the alveolar bone (Persson et al., 1998; Yagci et al., 2012). Thus, orthodontists should pay more attention to patients with a thin gingival biotype and a thin bone biotype (Evangelista et al., 2010). The influence of bone tissue on the occurrence of OGE is a reflection of tissue loss and accumulation, at least to a certain extent. However, the gingival biotype plays a decisive role in preventing gingival recession even in the presence of reduced or missing alveolar bone (Evangelista et al., 2010).

## Limitations

There are inherent limitations in this study that need to be considered. First, FEA limits the extrapolation of results to actual clinical situations. The positive association between gingival biotype and alveolar bone biotype doesn’t mean the measurement of the latter can stand for the result of the former. Additional clinical trials that evaluate the incidence and biomechanics of OGE in patients with different gingiva biotypes would facilitate our approach to clinical practice. Second, FEA cannot reflect the soft and hard tissue remodeling in the mouth, periodontal inflammation, and other complex clinical situation. Therefore, this method can play a supplementary role in finding the etiology of OGE, and the etiology of OGE needs more research. Finally, different tooth inclinations often correspond to different anterior alveolar morphologies. Models set up by changing the inclination of the anterior teeth may cause deviations to some extent. Therefore, it is difficult to simulate the personalized bone morphology of different patients by FEA; therefore, CBCT should prevail in clinical application.

## Conclusion

In this study, we investigated the biomechanics behind the occurrence of OGE in a new model through FEA. The biomechanics of OGE with different inclination angles in incisors and alveolar bone biotypes during the intrusion movement were analyzed in detail. As inclination increased or labial bone tissue became thinner, the surrounding tissues became more stressed and the extent of strain was greater in the OGE region. These findings highlight the fact that clinicians should pay more attention to patients with labial inclination or a thin bone biotype to prevent the occurrence of orthodontic complications such as OGE, root resorption, and bone dehiscence.

## Data Availability

The raw data supporting the conclusion of this article will be made available by the authors, without undue reservation.
